# The powerful synergistic effect of spiramycin/propolis loaded chitosan/alginate nanoparticles on acute murine toxoplasmosis

**DOI:** 10.1371/journal.pntd.0010268

**Published:** 2022-03-16

**Authors:** Nancy Abd-elkader Hagras, Nermine Mogahed Fawzy Hussein Mogahed, Eman Sheta, Amira Abd-elfattah Darwish, Mohamed Ali El-hawary, Moaaz Tarek Hamed, Bassma Hassan Elwakil

**Affiliations:** 1 Department of Medical Laboratory Technology, Faculty of Applied Health Sciences Technology, Pharos University in Alexandria, Alexandria, Egypt; 2 Department of Medical Parasitology, Faculty of Medicine, Alexandria University, Alexandria, Egypt; 3 Department of Pathology, Faculty of Medicine, Alexandria University, Alexandria, Egypt; 4 Department of Botany & Microbiology, Faculty of Science, Alexandria University, Alexandria, Egypt; JH-Institute of Molecular Medicine, INDIA

## Abstract

The novel formula of spiramycin/propolis loaded chitosan (CS)/alginate (Alg) nanoparticles (NPs) was assessed for *Toxoplasma gondii (T*. *gondii)* treatment in comparison with the commercially available spiramycin regarding tissue penetration and blood brain barrier (BBB) passage. Swiss Albino mice were inoculated intraperitoneally by 2500 tachyzoites of the virulent *T*. *gondii* RH strain. The experimental groups were treated with oral spiramycin, propolis, CS/Alg NPs, spiramycin loaded CS/Alg NPs, propolis loaded CS/Alg NPs, and spiramycin/propolis loaded CS/Alg NPs. The results demonstrated that spiramycin/propolis loaded CS/Alg NPs exerted the longest survival time with no mortality on the sacrifice day (8^th^) in addition to representing the highest significant parasite percent reduction of (≥96% reduction) in liver, spleen and brain designating successful tissue penetration and BBB passage. Tachyzoites treated with spiramycin/propolis loaded CS/Alg NPs demonstrated the most disfigured rapturing organism via scanning electron microscope examination along with representing an overall remarkable improvement of the histopathological pictures of liver, spleen and brain. In conclusion, spiramycin/propolis loaded CS/Alg NPs showed the uppermost efficacy in the treatment of acute murine toxoplasmosis. The safe nature and the anti-parasitic effect of each of CS, Alg, spiramycin and propolis encourage the synergistic use of spiramycin/propolis loaded CS/Alg NPs as a potent treatment for human toxoplasmosis.

## Author summary

Toxoplasmosis is a disease that results from infection with a parasite known as *Toxoplasma gondii* (*T*. *gondii*). The parasite is considered to be one of the world’s most common parasites. Infection frequently occurs by eating undercooked contaminated meat, exposure to infected cat faeces, or mother-to-fetus transmission throughout pregnancy. The parasite has the ability to reach all body organs including the brain. This in turn, may lead to death specially among people with weakened immune systems and infants born to infected mothers. There are many treatment options for the disease, however they may have severe side effects or can’t fully reach the brain. Consequently, the present work focused on the formulation of a safe novel formula at the nano size for better tissue penetration. The novel treatment was achieved by preparing the natural organic chitosan/alginate nanoparticles and loading them with spiramycin (the safest drug used in toxoplasmosis) along with propolis (a natural bee product). The treatment efficacy of the novel spiramycin/propolis loaded chitosan/alginate nanoparticles was compared with the commercial spiramycin. The used drug showed a marked potency in toxoplasmosis treatment. The anti-parasitic effect and the safe nature of each of the used components potentiate its use as a promising treatment.

## Introduction

*Toxoplasma gondii* (*T*. *gondii*) is a highly prevalent zoonotic parasite accountable for the infection of about half of the world’s population [1,2]. The parasite is single-celled that is capable of infecting any nucleated cell of almost all warm-blooded animals and birds [3]. *T*. *gondii* is an obligate intracellular parasite which can’t survive without a host owning a complex life cycle alternating between sexual and asexual reproductive cycles [4]. Each cycle takes place in a different host where the sexual cycle occurs in the feline definitive host, whereas the asexual cycle occurs in any warm-blooded intermediate host [2]. *T*. *gondii* owes three different stages in its life cycle; the rapidly proliferative tachyzoite, the slowly proliferative bradyzoite which is the encysted stage found in tissues and the oocyst which is the sexual stage found in the intestine of feline [3]. Human can be infected by many sources; ingestion of undercooked meat contaminated with tissue cysts, ingestion of unclean vegetables or water contaminated with cats’ oocysts, or by placental transfer from an infected mother to her fetus[2]. The infection with toxoplasma is categorised into acute and chronic stages. The rapidly dividing tachyzoites initiate the acute stage where they multiply intracellularly causing host cell rupture. Tachyzoites penetrate nearly all tissues including liver, spleen, brain, muscles, eyes, placenta and lungs [5]. Tachyzoites of the highly virulent toxoplasma strains (e.g. RH) are known for their lethality without tissue cyst (bradyzoites) formation [6]. Although the infection may be asymptomatic, drastic pathology and lethality are prevalent outcomes in immunocompromised individuals or congenitally infected infants [2].

Despite the variety of the currently available toxoplasma drugs, unluckily they don’t act strongly with toxoplasmosis because of their side effects or their poor penetration of the blood brain barrier (BBB). Consequently, there is a critical necessity to attain new treatment options possessing effectiveness and safety to humans [2,7]. Pyrimethamine-sulfadiazine is used as the first-line therapy, however pyrimethamine affects the bone marrow negatively leading to the reduction of the patient’s immune response even when taken with folinic acid. In patients who have sulfa allergy, sulfadiazine can be replaced with clindamycin which is an antibiotic that yet have similar levels of toxicity as well [2,7]. Pyrimethamine-sulfadiazine can also be replaced by trimethoprim-sulfamethoxazole when pyrimethamine is unavailable [8]. Other treatment options can be used as atovaquone or azithromycin when the first-line therapy is not well tolerated. Nevertheless, they are supported by fewer clinical data and they have the same rates of patient intolerance [2]. Use of spiramycin is another treatment alternative [9]. Studies revealed that it has potential effectiveness against acute toxoplasmosis, have less toxicity and achieve higher concentrations in the placenta than other drugs helping in the prevention of parasite transmission from the mother to fetus during pregnancy [2]. Despite of the marvellous advantages of spiramycin, yet it poorly penetrates the BBB. Thus, there is a crucial need for further development of spiramycin in order to benefit from its advantages [2,5,10].

In the search for natural treatment options, propolis also known as bee glue, is a natural material collected by bees from the leaf buds of a wide variety of tree types such as pine and palm [11]. Propolis is used in coating the hive walls to create a smooth germfree surfaces for the attachment of honeycombs in addition to the aseptic filling of gaps to prevent the entrance of any pathogenic agent [12]. Propolis is very complex as it contains more than 180 compounds, mainly comprising flavonoids accompanied by phenolic acid and ester [13]. Since ancient times, propolis was used in many fields as antiseptic wound treatment and mummification process due to its anti-rot properties. Modern research proved that propolis owes a significant anti-bacterial, anti-fungal, anti-viral, hepatoprotective, anti-oxidant, anti-inflammatory and immunomodulatory properties [11].

Nowadays, the practice of nanotechnology is becoming a common approach in the treatment of many diseases. Successful drug delivery is ensured by nanotechnology via improving the drug bioavailability and the drug permeability through membranes, consequently lowering the drug doses required [14]. Nanotechnology focuses on formulating biocompatible polymeric nanoparticles (NPs) that are capable of bypassing biological barriers such as BBB [15]. Chitosan (CS) is an attention-grabbing natural linear cationic polysaccharide polymer, approved by the Food and Drugs Administration (FDA). It is obtained from the deacetylation of chitin which is abundantly present in the exoskeleton of crustaceans. CS NPs have perfect biodegradable and biocompatible properties which make it an ideal choice for drug delivery besides its great anti-microbial and anti-parasitic action Marques [16]. Sodium alginate (Alg) is another FDA approved natural linear anionic polysaccharide polymer. It is composed of sodium salt of alginic acid which is mainly found in the cell wall of green algae [17]. Alg NPs are characterized by their biocompatibility and their ability of conversion from liquid to gel in aqueous medium which potentiate its use as antacid and anti-microbial wound dressing [18].

In the present study, the therapeutic effectiveness of spiramycin, propolis, CS/Alg NPs, spiramycin loaded CS/Alg NPs, propolis loaded CS/Alg NPs and the novel spiramycin/propolis loaded CS/Alg NPs has been assessed in murine model infected with the highly virulent toxoplasma RH strain.

## Materials and methods

### I. Ethics statement

The protocol of the present study was approved by the Ethics Committee of the Faculty of Medicine, Alexandria University (0104837) following the international regulations of animal care.

### II. *T*. *gondii* strain

*T*. *gondii* RH virulent strain was used in this study. The strain was acquired from the Parasitology Department, Faculty of Medicine, Alexandria University, Egypt. Maintenance of the strain was attained via serial intraperitoneal passage of tachyzoites in Swiss Albino mice. On the 4^th^ day post-infection, mice were sacrificed and their peritoneal fluid was washed with saline. Using the haemocytometer, a drop of the peritoneal fluid was used to adjust the tachyzoites count for mice infection at a dose of 2500 tachyzoites/100 μl saline / mouse [2].

### III. The used drugs

The used drugs were spiramycin, propolis, chitosan (CS)/alginate (Alg) nanoparticles (NPs), spiramycin loaded CS/Alg NPs, propolis loaded CS/Alg NPs, and spiramycin/propolis loaded CS/Alg NPs. All the used treatments were orally administered from day zero of infection for 7 days.

#### III.1. Preparation of drugs

*III*.*1*.*1*. *Spiramycin preparation*. The dose of spiramycin (Unipharma Pharmaceutical Company) per mouse was calculated and dissolved in 100 μl saline for oral administration [2].

*III*.*1*.*2*. *Propolis preparation*. Propolis sample was collected during autumn 2020 from Alexandria, Egypt. Propolis sample (20% w*/*v) was grinded to powder and extracted using ethanol (99.9%) by maceration in dark glass container with continuous stirring then sonicated for one hour, filtered and stored in dark container at 4°C until further use. The propolis full spectra was analysed using GC-MS analysis which has been reported in details in our previously reported work [19].

*III*.*1*.*3*. *CS/Alg NPs preparation*. Both CS and Alg were purchased from Sigma-Aldrich (MO, USA). CS/Alg NPs were prepared using ionic gelation method according to Thai et al. (2020). Alg solution (1% w/v) was prepared and the solution viscosity was increased by adding CaCl2 (0.05 M) drop-wisely. CS solution (1% w/v) was prepared by dissolving CS powder in acidified water (1% acetic acid v/v). Alg solution was added to CS solution drop-wisely and stirred in a magnetic stirrer till solution became homogenous for 15 minutes. The ratio of Alg to CS was kept 62.2 to 28.8% in all the preparations [20].

*III*.*1*.*4*. *Spiramycin loaded CS/Alg NPs preparation*. Spiramycin loaded CS/Alg NPs were prepared in accordance with Hagras et al. (2019) with some modifications. Alg and CS solutions were prepared as previously mentioned. Eight ml of spiramycin solution (containing 900 mg spiramycin) were mixed with the Alg solution. The mixture was then added to the freshly prepared CS solution drop-wisely then kept under stirring condition for 15 minutes [2,20].

*III*.*1*.*5*. *Propolis loaded CS/Alg NPs preparation*. Propolis loaded CS/Alg NPs were prepared by ionic gelation method according to Hegazi et al. (2019) with some modifications. Alg and CS solutions were prepared as previously mentioned. Eight ml of filtered propolis extract (containing 355 mg propolis) were mixed with the alginate solution. The mixture was then added to the freshly prepared CS solution drop-wisely then kept under stirring condition for 15 minutes [20,21].

*III*.*1*.*6*. *Spiramycin/propolis loaded CS/Alg NPs preparation*. The preparation of spiramycin/propolis loaded chitosan/alginate nanoparticles was a novel formulation as well as the combination between spiramycin and propolis. Alg and CS solutions were prepared as previously mentioned. Four ml of spiramycin solution (containing 900 mg spiramycin) were mixed with four ml of propolis extract (containing 355 mg propolis) then the mixture was added to the Alg solution. The mixture was then added to the freshly prepared CS solution drop-wisely then kept under stirring condition for 15 minutes.

#### III.2. Nanoparticles characterization

Each of the prepared NPs formulations was examined by scanning electron microscope (SEM, JOEL, JSM-5300, Japan) and transmission electron microscope (TEM, JEOL, JSM- 2100 Plus, Japan) to assess the size, surface and shape of the prepared NPs. The zeta potential (ζ) of the NPs was determined by the dynamic light scattering (DLS) technique using Malvern Zetasizer [2,22]. Determination of the entrapment efficiency was assessed. The prepared nanoparticles were diluted (1:10 v*/*v) with phosphate-buffer solution and then centrifuged at 15,000 rpm for 15 min at 4°C. Supernatants were measured at 260 nm by UV*/*Vis (Spekol 1300, Analytik Jena, Germany) with methanol as a blank to measure the unentrapped drug [22,23]. The entrapment efficiency percentage (EE%) was calculated according to the following equation:

$$EE\%=\frac{Total\;unentrapped\;drug\;}{Total\;drug}\times 100$$

### IV. Experimental design (Fig 1)

Seventy-five laboratory male Swiss Albino mice were obtained from the animal house of Pharos University in Alexandria, Egypt. At the beginning of the experiment, each mouse was 6–8 weeks old, weighing 20–25 g. Except the uninfected control mice, each mouse was infected intraperitoneally with the RH strain in dose of 2500 tachyzoites/100 μl [2].

Mice were divided into the following groups:

- **Group I:** Control group (15 mice):
- **Subgroup Ia (5 mice):** Uninfected control which was used for the assessment of mice clinical picture, survival time and mortality rate [2].
- **Subgroup Ib (10 mice):** Infected untreated control where each mouse received 100 μl saline (the vehicle used for drugs suspension) orally by gavage needle from the start of infection for seven days [2,24].
- **Group II:** Experimental group (60 mice):
- **Subgroup IIa (10 mice):** Infected mice treated with spiramycin in a dose of 400 mg/kg/day [2,10].
- **Subgroup IIb (10 mice)**: Infected mice treated with propolis in a dose of 150 mg/kg/day[19,25].
- **Subgroup IIc (10 mice):** Infected mice treated with CS/Alg NPs in a dose of 400 mg/kg/day [20].
- **Subgroup IId (10 mice)**: Infected mice treated with spiramycin loaded CS/Alg NPs in a dose of 400 mg/kg/day [2].
- **Subgroup IIe (10 mice)**: Infected mice treated with propolis loaded CS/Alg NPs in a dose of 150 mg/kg/day.
- **Subgroup IIf (10 mice)**: Infected mice treated with spiramycin/propolis loaded CS/Alg NPs in a dose of 550 (400 spiramycin/150 propolis) mg/kg/day.
- All those formulae were suspended in 100 μl saline/dose/mouse and then administered orally by gavage needle from the start of infection for seven days [2].
- Five mice of each subgroup (controls and experimental) were daily observed to govern their clinical picture and survival time until their death.
- The other five mice were ethically anesthetized and sacrificed by cervical dislocation on the 8th day post-infection (24 h after the last dose of treatment). From the infected control subgroup (subgroup Ib) and the six treated subgroups (subgroups IIa-IIf); liver, spleen and brain were obtained for further parasitological studies. Morphological study using scanning electron microscope (SEM) was performed using the glutaraldehyde-fixed peritoneal fluid of each mouse. Tissue sections of liver, spleen and brain were used for histopathological tissue investigation.

**Fig 1:**
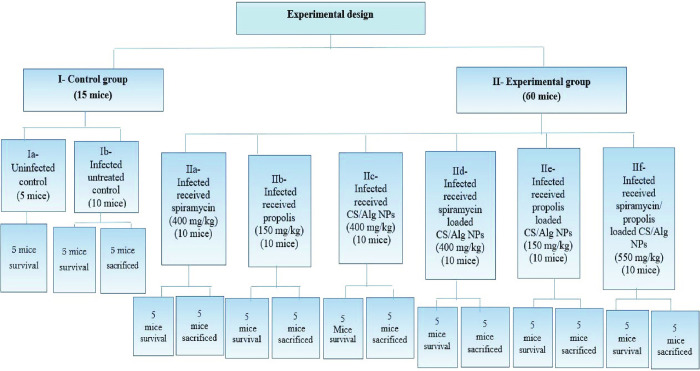
Experimental design of the studied groups.

### V. Evaluation of the drug efficacy

Clinical, parasitological, morphological and histopathological studies were used in the assessment of drug efficacy as follows:

### V.1. Clinical study

Daily observation of mice was done to spot any changes in clinical behaviour (posture and activity) and food intake [26].

### V.2. Parasitological study

*V*.*2*.*1*. *Survival time*. Kaplan-Meier survival curve was used for daily observation of mice (5 mice/ each studied subgroup) for 30 days to determine the percentage of mice living over time [24,27].

*V*.*2*.*2*. *Mortality rate (MR) on the 8*^*th*^ *day post-infection*. The mortality rate (MR) on the 8th day post-infection of all subgroups was calculated as it was estimated to be the maximum survival suspected for infected untreated control. MR was calculated according to the equation: [2]

$$MR\;(\%)=\frac{Number\;of\;dead\;mice\;at\;the\;sacrifice\;time}{Number\;of\;mice\;at\;the\;beginning\;of\;the\;experimental}x\;100$$

*V*.*2*.*3*. *Parasite burden*. Impression smears of liver, spleen and brain were performed followed by their staining with Giemsa stain. Tachyzoites were counted in the stained slides and the mean count of twenty different oil immersion fields from each organ of each mouse was then attained (ten fields/each slide and two slides/each organ). The mean number of tachyzoites in each subgroup of mice was then calculated [26].

*V*.*2*.*4*. *Parasite percent reduction (%R)*. Calculation of the % R in the parasite count in liver, spleen or brain was estimated by using the equation: [2,26]

$$\%R=\frac{Mean\;count\;in\;infected\;untreated\;control\;group–Mean\;count\;in\;experimental\;group}{Mean\;count\;in\;infected\;untreated\;control\;group}x\;100$$

### V.3. Morphological study by scanning electron microscope (SEM)

On the sacrifice day, the peritoneal exudate was fixed in glutaraldehyde and prepared for further examination of tachyzoites ultra-structure by SEM [2].

### V.4. Histopathological study

Tissue samples from liver, spleen and brain were individually collected from the different groups and fixed in 10% of neutral formalin, sectioned and stained with hematoxylin-eosin. The stained slides were examined by histopathology using Olympus binocular microscope (CX21/LED) to assess histologic alteration in different groups [5,28,29].

### VI. Statistical analysis of the data

Data were fed to the computer and analyzed using IBM SPSS software package version 20.0. **(**Armonk, NY: IBM Corp**).** The Kolmogorov- Smirnov was used to verify the normality of distribution of variables. ANOVA was used for comparing the different studied groups and followed by Post Hoc test (Tukey) for pairwise comparison. Kaplan-Meier Survival curve was used for overall survival. Comparisons between groups for categorical variables were assessed using Chi-square test (Fisher’s Exact or Monte Carlo correction). Significance of the obtained results was judged at the 5% level [30].

## Results

### I. Characterization of nanoparticles (NPs)

#### I.1. Scanning electron microscope (SEM) and transmission electron microscope (TEM) analyses of the prepared NPs

**Fig 2:**
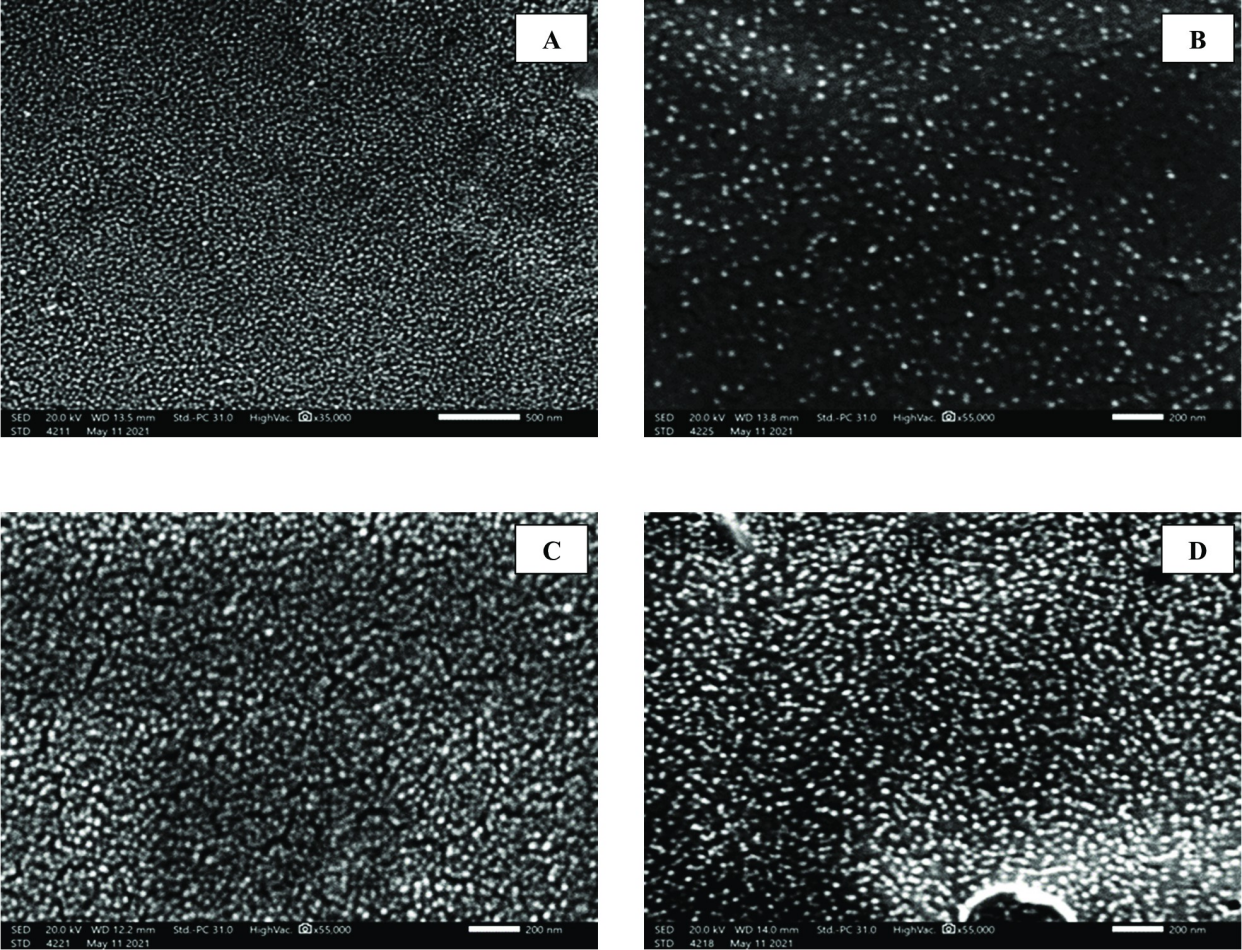
SEM micrographs of the prepared NPs showing regular, smooth and spherical shape. (A) CS/Alg NPs (X 35,000). (B) Spiramycin loaded CS/Alg NPs (X 55,000). (C) Propolis loaded CS/Alg NPs (X 55,000). (D) Spiramycin/propolis loaded CS/Alg NPs (X 55,000).

**Fig 3:**
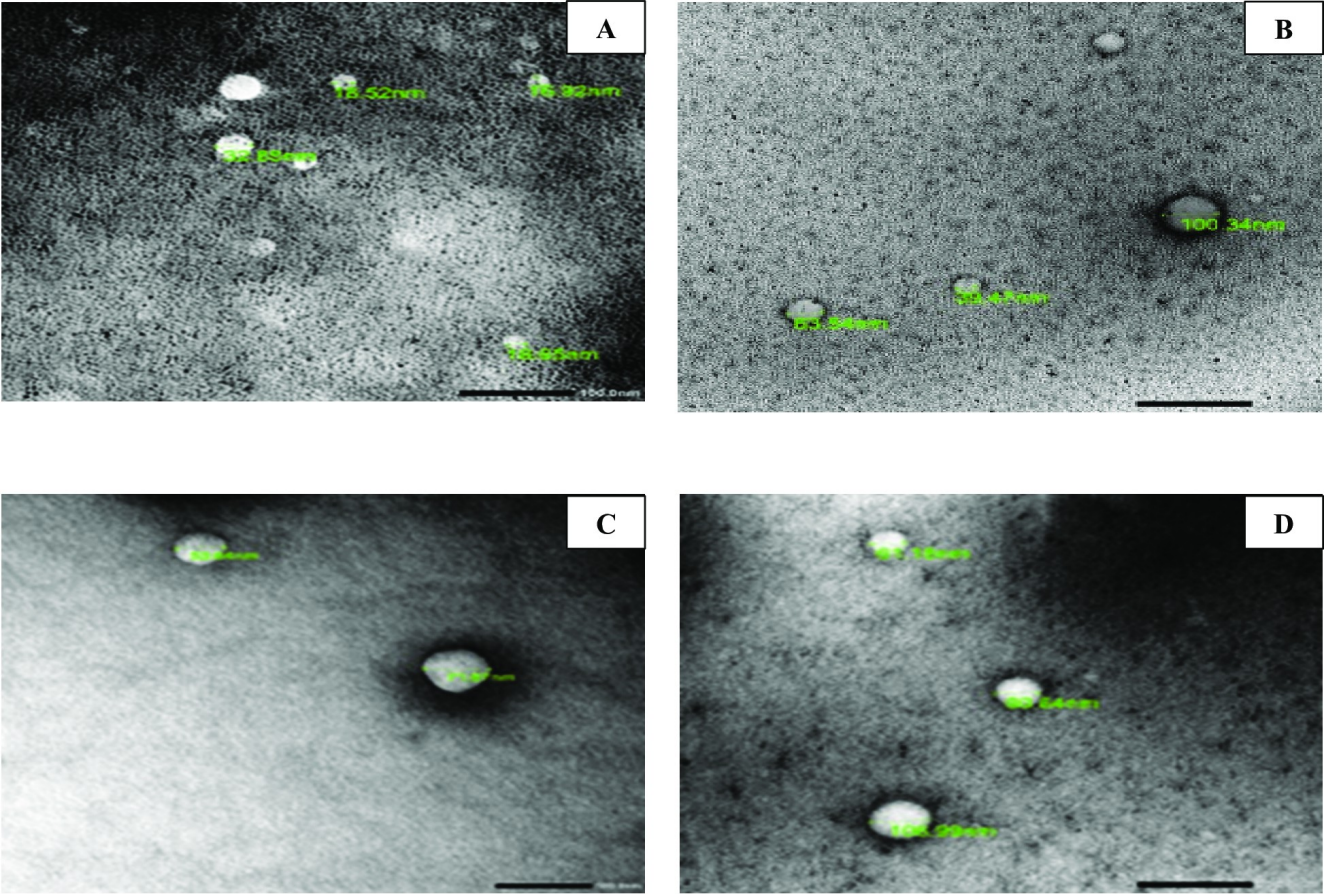
TEM micrographs of the prepared NPs revealing increase in particle size upon drug loading (X 55,000). (A) CS/Alg NPs. (B) Spiramycin loaded CS/Alg NPs. (C) Propolis loaded CS/Alg NPs. (D) Spiramycin/propolis loaded CS/Alg NPs.

Data in Figs 2 and 3 revealed that the prepared NPs had a regular, smooth and spherical shape with successful increase in particle size upon drug loading. The average particle sizes were 20, 58, 62 and 67.3 nm for CS/Alg NPs, spiramycin loaded CS/Alg NPs, propolis loaded CS/Alg NPs and spiramycin/propolis loaded CS/Alg NPs respectively.

#### I.2. Zeta-potential and entrapment efficiency measurements

Data in Table 1 revealed that the loaded NPs had a positive charge reflecting the stability of the prepared formulae while CS/Alg NPs (placebo) had a negative charge which may be attributed to the nature of the loaded Alg. The entrapment efficiency was evaluated for the loaded spiramycin. It was noticed that the added core shell of propolis enhanced the entrapment efficiency of spiramycin (85.1%) compared to spiramycin loaded CS/Alg NPs (79.3%).

**Table 1:** Zeta-potential (ζ) and entrapment efficiency (EE) of the prepared nano formulae.

Formulations	ζ potential (mV)	EE%
CS/Alg NPs	-25.7	-
Spiramycin loaded CS/Alg NPs	+35.1	79.3
Propolis loaded CS/Alg NPs	+38.7	-
Spiramycin/propolis loaded CS/Alg NPs	+35.6	85.1

## II. Evaluation of the drug efficacy

### II.1 Clinical study

On the fifth day post-infection, there was a marked reduction of food intake among infected untreated control along with decreased activity, hunched posture and ruffled fur compared to normal mice. On the other hand, infected treated mice showed normal food intake with better activity. Spiramycin/propolis loaded CS/Alg NPs treated subgroup appeared remarkably healthier than all other subgroups.

#### II.2. Parasitological study

**Fig 4:**
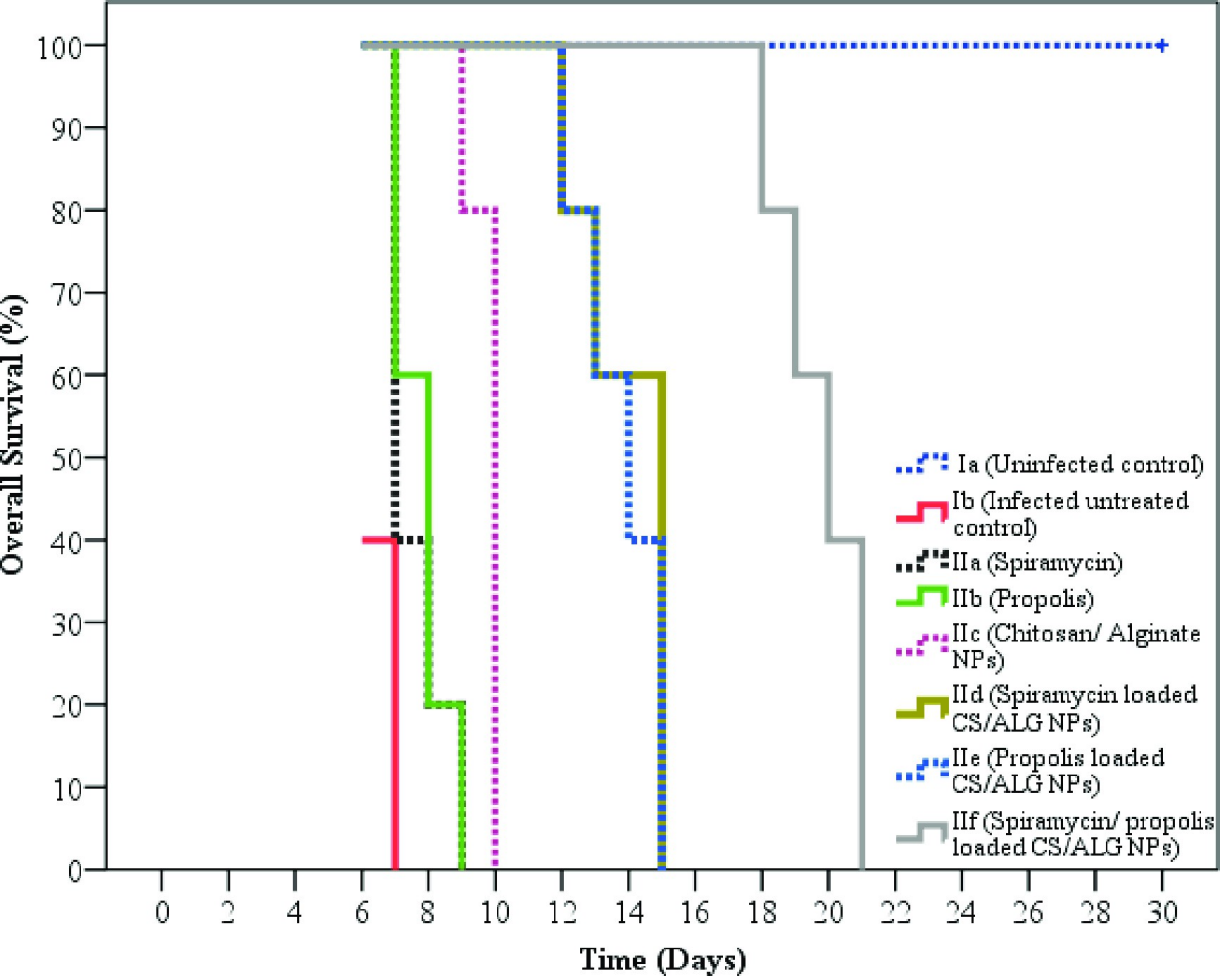
Kaplan-Meier overall survival curve for all subgroups.

*II*.*2*.*1*. *Survival time*. The survival time was assessed for 30 days. Statistically, there was a high significant difference in mice survival between all groups (p < 0.001). As the tremendous difference is clearly shown in the mean survival time, the uninfected mice had a mean time of 30.0 days, whereas infected untreated mice showed a mean time of 6.4 days. Regarding treated mice, they flaunted a mean survival time ranging between 7.6 and 19.8 days. The most extended survival time was observed among mice receiving spiramycin/propolis loaded CS/Alg NPs. (Table 2 and Fig 4)

**Table 2:** Comparison between all subgroups regarding mice mean survival time and mortality rate on the eighth day post-infection.

	Subgroup Ia (Uninfected control)	Subgroup Ib (Infected untreated control)	Subgroup IIa (Spiramycin)	Subgroup IIb (Propolis)	Subgroup IIc (CS/Alg NPs)	Subgroup IId (Spiramycin loaded CS/Alg NPs)	Subgroup IIe (Propolis loaded CS/Alg NPs)	Subgroup IIf (Spiramycin/propolis loaded CS/Alg NPs)
**Survival time**								
Mean ± SD.	30.0 ± 0.0	6.4±0.55	7.6±0.89	7.8± 0.84	9.8±0.45	14.0±1.41	13.8±1.3	19.8±1.3
**χ**^**2**^ **(p)**	75.581* (<0.001*)							
**p**_**1**_		0.002*	0.002*	0.002*	0.003*	0.003*	0.003*	0.003*
**p**_**2**_			0.031*	0.018*	0.002*	0.002*	0.002*	0.002*
**p**_**3**_				0.770	0.004*	0.002*	0.002*	0.002*
**p**_**4**_					0.005*	0.002*	0.002*	0.002*
**p**_**5**_						0.003*	0.003*	0.003*
**p**_**6**_							0.637	0.003*
**p**_**7**_								0.003*
**Mortality rate (%) on 8**^**th**^ **day**	0.0	100.0	80.0	80.0	0.0	0.0	0.0	0.0
**χ**^**2**^ **(**^**MC**^**p**_**0**_**)**	28.551* (<0.001*)							
^**FE**^ **p**_**1**_		0.008*	0.048*	0.048*	–	–	–	–
^**FE**^ **p**_**2**_			1.000	1.000	0.008*	0.008*	0.008*	0.008*
^**FE**^ **p**_**3**_				1.000	0.048*	0.048*	0.048*	0.048*
^**FE**^ **p**_**4**_					0.048*	0.048*	0.048*	0.048*
**p**_**5**_						–	–	–
**p**_**6**_							–	–
**p**_**7**_								–

χ^2^: **Chi square test** MC: **Monte Carlo** FE**: Fisher Exact**

p: p value for **Log rank test** from **Kaplan-Meier** for comparing between the studied groups

p_0_: p value for comparing between the studied groups

p_1_: p value for comparing between **Subgroup Ia** and each other group

p_2_: p value for comparing between **Subgroup Ib** and each other group

p_3_: p value for comparing between **Subgroup IIa** and each other group

p_4_: p value for comparing between **Subgroup IIb** and each other group

p_5_: p value for comparing between **Subgroup IIc** and each other group

p_6_: p value for comparing between **Subgroup IId** and each other group

p_7_: p value for comparing between **Subgroup IIe** and **Subgroup IIf**

*: Statistically significant at p ≤ 0.05

*II*.*2*.*2*. *Mortality rate (MR) on the 8*^*th*^ *day post-infection*. On the eighth day post-infection, no mice died among the uninfected control while the MR was 100% in the infected untreated control. The MR was 80% among both spiramycin and propolis treated subgroups. On the other hand, no mice died among all NPs treated subgroups including CS/Alg NPs, spiramycin loaded CS/Alg NPs, propolis loaded CS/Alg NPs and spiramycin/propolis loaded CS/Alg NPs. (Table 2)

**Fig 5:**
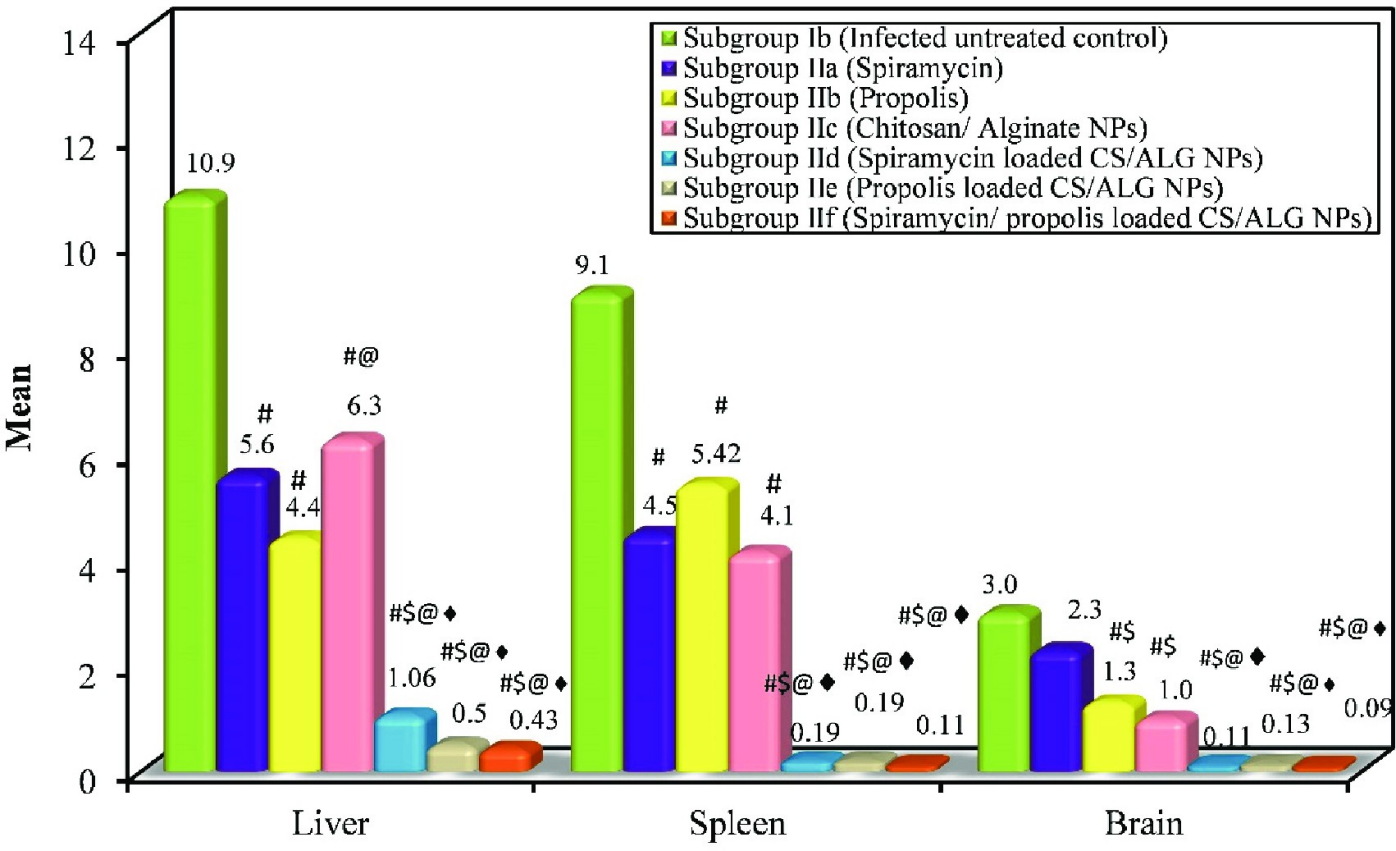
Comparison between the different studied subgroups according to the mean parasite count in liver, spleen and brain.

**Fig 6:**
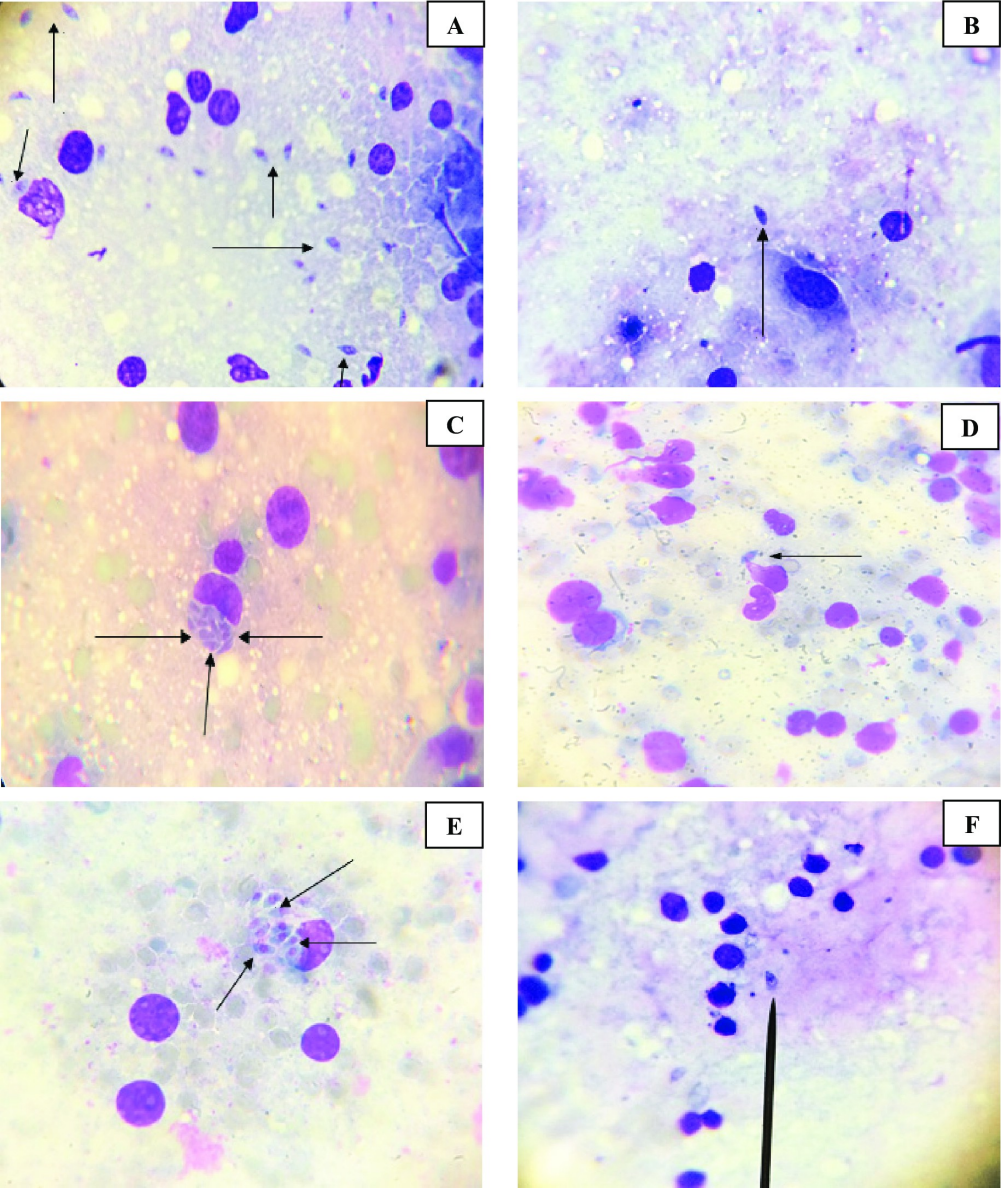
Giemsa-stained impression smears of liver, spleen and brain revealing *T*. *gondii* tachyzoites. (A) *Toxoplasma* tachyzoites in infected untreated control liver (Giemsa stain X 1000). (B) *Toxoplasma* tachyzoites in infected treated liver (Giemsa stain X 1000). (C) *Toxoplasma* tachyzoites in infected untreated control spleen (Giemsa stain X 1000). (D) *Toxoplasma* tachyzoites in infected treated spleen (Giemsa stain X 1000). (E) *Toxoplasma* tachyzoites in infected untreated control brain (Giemsa stain X 1000). (F) *Toxoplasma* tachyzoites in infected treated brain (Giemsa stain X 1000).

*II*.*2*.*3*. *Parasite burden and percent reduction (%R)*. Statistically, there was a high significant difference in parasite count between all groups (ANOVA test, p < 0.001). The mean tachyzoites count in the liver, spleen and brain of the infected untreated control was 10.88, 9.06 and 3.0/ 20 oil immersion fields respectively. Regarding the treated subgroups, the lowest mean parasite count (i.e. highest percent of reduction) was obtained among mice treated with the spiramycin/propolis loaded CS/Alg NPs. (Table 3 and Figs 5 and 6).

**Table 3:** Parasite count and percent reduction in liver, spleen and brain among the different studied subgroups.

	Subgroup Ib (Infected untreated control)	Subgroup IIa (Spiramycin)	Subgroup IIb (Propolis)	Subgroup IIc (CS/Alg NPs)	Subgroup IId (Spiramycin loaded CS/Alg NPs)	Subgroup IIe (Propolis loaded CS/Alg NPs)	Subgroup IIf (Spiramycin/propolis loaded CS/Alg NPs)
**Liver**	**m(n = 5)**	**(n = 5)**	**(n = 5)**	**(n = 5)**	**(n = 5)**	**(n = 5)**	**(n = 5)**
Mean	10.88	5.60^#^	4.40^#^	6.28^#^^@^	1.06^#^^$^^@^^♦^	0.50^#^^$^^@^^♦^	0.43^#^^$^^@^^♦^
±SD.	1.78	0.96	0.42	0.19	0.74	0.43	0.24
**F (p)**	101.477* (<0.001*)						
**% Reduction**		**48.5**	**59.6**	**42.3**	**90.3**	**95.4**	**96.0**
**Spleen**							
Mean	9.06	4.50^#^	5.42^#^	4.14^#^	0.19^#^^$^^@^^♦^	0.19^#^^$^^@^^♦^	0.11^#^^$^^@^^♦^
±SD.	1.93	0.45	0.40	0.21	0.12	0.09	0.11
**F (p)**	97.101*(<0.001*)						
**% Reduction**		**50.3**	**40.2**	**54.3**	**97.9**	**97.9**	**98.8**
**Brain**							
Mean	3.0	2.30	1.30^#$^	1.0^#$^	0.11^#$^^@^^♦^	0.13^#$^^@^^♦^	0.09^#$^^@^^♦^
±SD.	0.16	0.60	0.71	0.54	0.11	0.10	0.08
**F (p)**	38.834*(<0.001*)						
**% Reduction**		**23.3**	**56.7**	**66.7**	**96.3**	**95.7**	**97.0**

Data was expressed using **Mean ± SD**.

**F**: **F for ANOVA test**, Pairwise comparison bet. each 2 groups was done using **Post Hoc Test (Tukey)**

p: p value for comparing between the studied groups

*: Statistically significant at p ≤ 0.05

#: Significant with **Subgroup Ib** $: Significant with **Subgroup IIa**

@: Significant with **Subgroup IIb** ♦: Significant with **Subgroup IIc**

♠: Significant with **Subgroup IId** ♣: Significant with **Subgroup IIe**

### II.3. Morphological study

**Fig 7:**
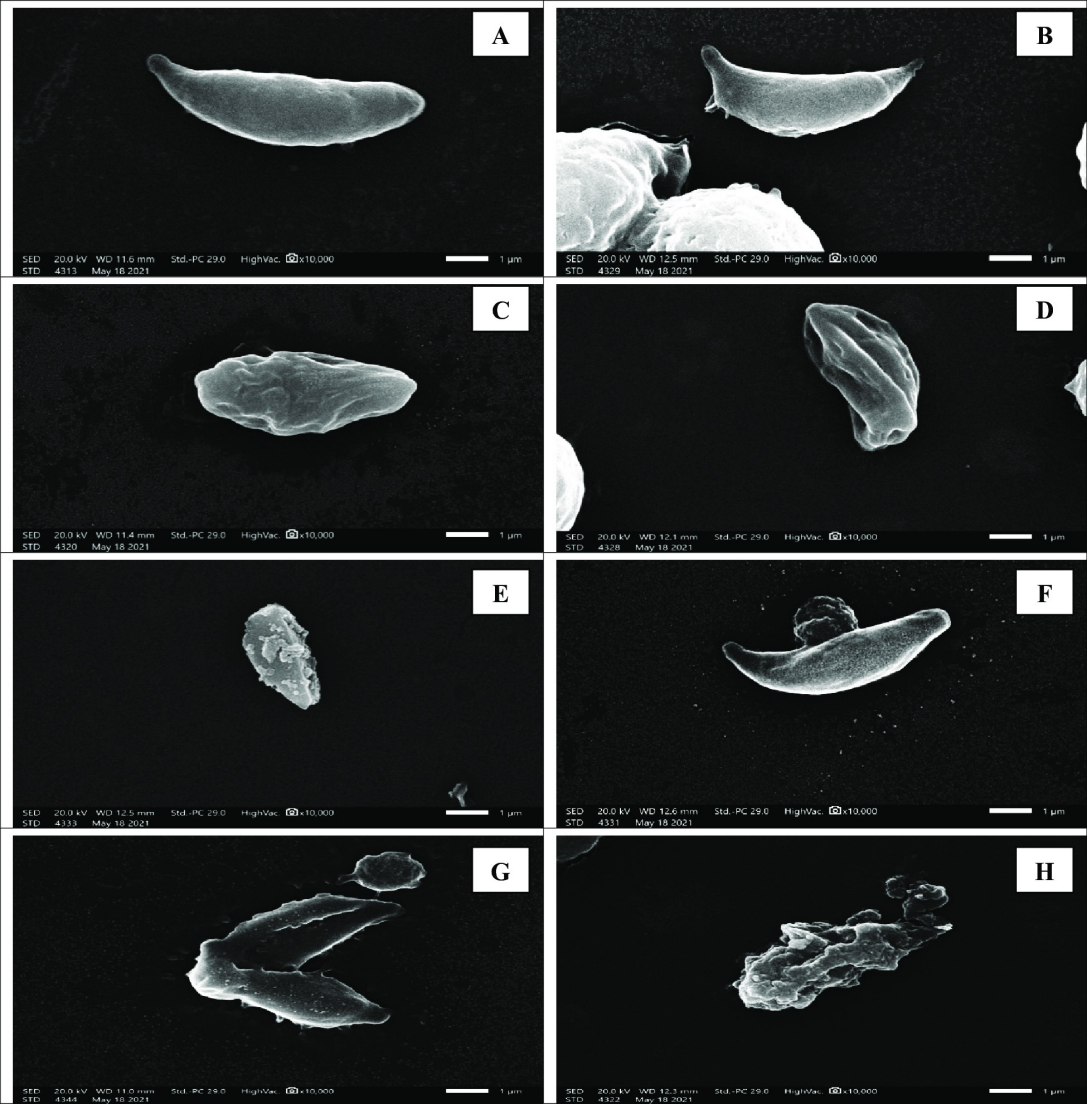
SEM of *Toxoplasma* tachyzoites. (A) Tachyzoite from subgroup I b (infected untreated control), revealing crescent shape with apparent conoid (X 10,000). (B) Tachyzoite from subgroup II a, showing evident loss of its smooth surface with erosion and protrusion of the surface. (X 10,000). (C) Tachyzoite from subgroup II b, showing shrunken organism losing its crescent shape, smooth surface, conoid and tapered ends with profound furrows and ridges (X 10,000). (D) Tachyzoite from subgroup II c, showing a shrunken distorted parasite losing the crescent shape, tapered ends and conoid with noticeable surface erosion and ulceration (X 10,000). (E) Tachyzoites from subgroup II d, showing loss in the crescent shape, smooth surface, conoid and tapered ends with multiple blebs on the parasite surface (X 10,000). (F) Tachyzoites from subgroup II e, showing a rupturing parasite losing its smooth surface (X 10,000). (G) and (H) Tachyzoites from subgroup II f, showing disfigured rupturing organism losing its conoid with disorganised lacerated surface (X 10,000).

SEM was used to study the morphology of tachyzoites. Tachyzoites in the peritoneal fluid of infected untreated mice were smooth in surface, elongated, often crescent-shaped. They almost had round pole at one end and pointed pole at the other with obvious conoid. Nevertheless, tachyzoites collected from all treated subgroups revealed distortion with loss of smooth surface, erosions, protrusions, ulcerations, furrows, ridges and/or blebs. This was more apparent in mice treated with spiramycin/propolis loaded CS/Alg NPs where the tachyzoites appeared remarkably disfigured and rupturing (Fig 7).

### II.4. Histopathological study

#### II.4.A. Liver (Fig 8)

Histopathological examination of hepatic tissues of infected untreated group showed massive oedema of liver capsule with inflammatory exudate rich in parasite. All portal tracts showed moderate to severe inflammatory infiltrate with interface hepatitis. The infiltrate was composed mainly of lymphocytes and plasma cells. Marked vascular dilatation and congestion were seen in all hepatic vessels including central and portal veins as well as sinusoids. Large foci of lobular mononuclear infiltrates were spotted frequently with numerous intra and extra hepatocellular parasites.

**Fig 8:**
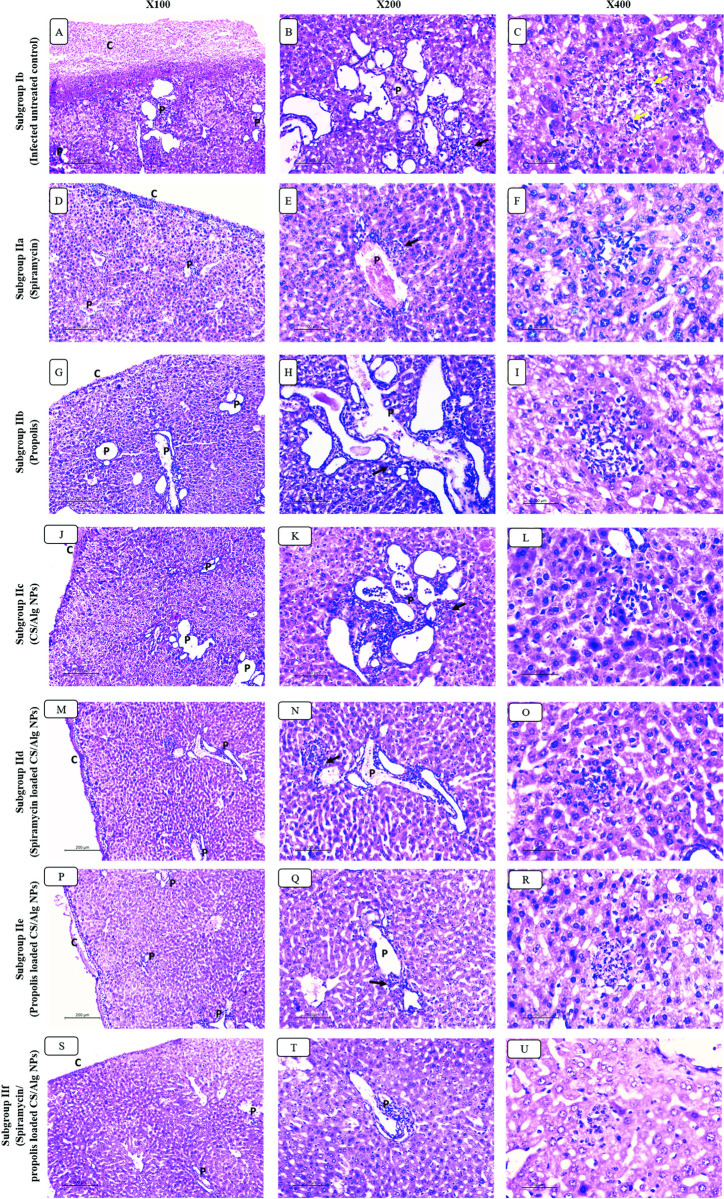
H&E-stained sections of hepatic tissues in different studied groups. Infected untreated group: (A) Severe capsular oedema and dense inflammatory exudate is seen on liver capsule (c) and diffuse severe portal inflammation (P). (B) Portal tract (p) showing severe mononuclear inflammation with interface hepatitis (black arrow) and marked vascular congestion. (C) Large mononuclear lobular inflammation focus showing necrotic hepatocytes and numerous parasites (yellow arrow). Spiramycin treated group: (D) Moderate capsular oedema with mild inflammatory exudate is seen on liver capsule (c) and moderate inflammation is seen in portal tracts (P). (E) Portal tract (p) showing moderate mononuclear inflammation with interface hepatitis (black arrow) and moderate vascular congestion. (F) Mononuclear lobular inflammation showing necrotic hepatocytes. Propolis treated group: (G) Moderate capsular oedema with mild inflammatory exudate is seen on liver capsule (c) and moderate/severe inflammation is seen in portal tracts (P). (H) portal tract (p) showing moderate mononuclear inflammation with interface hepatitis (black arrow) and marked vascular congestion. (I) Large mononuclear lobular inflammation focus showing necrotic hepatocytes. CS/Alg NPs treated group: (J) Moderate capsular oedema with mild inflammatory exudate is seen on liver capsule (c). Moderate/severe inflammation is seen in portal tracts (P). (K) Portal tract (p) showing moderate mononuclear inflammation with interface hepatitis (black arrow) and marked vascular congestion. (L) Large mononuclear lobular inflammation focus showing necrotic hepatocytes. Spiramycin CS/Alg NPs treated group: (M) Mild capsular oedema with no exudate on liver capsule (c). Moderate inflammation is seen in portal tracts (P). (N) Portal tract (p) showing moderate mononuclear inflammation with focal interface hepatitis (black arrow) and moderate vascular congestion. (O) Small mononuclear lobular inflammation focus showing few necrotic hepatocytes. Propolis CS/Alg NPs treated group: (P) Mild capsular oedema with no exudate on liver capsule (c). Minimal inflammation is seen in portal tracts (P). (Q) Portal tract (p) showing mild mononuclear inflammation with focal interface hepatitis (black arrow) and moderate vascular congestion. (R) Small mononuclear lobular inflammation focus showing few necrotic hepatocytes. Spiramycin/Propolis loaded CS/Alg NPs treated group: (S) Normal hepatic capsule with no oedema (c). Minimal inflammation is seen in rare portal tracts (P). (T) Portal tract (p) showing minimal mononuclear inflammation with no interface hepatitis. No vascular congestion is seen. (U) Small mononuclear lobular inflammation focus is occasionally seen.

Spiramycin, propolis and CS/Alg NPs treated infected models showed mild improvement of hepatic histology. Capsular oedema was slightly improved however most of the portal tracts showed moderate infiltration with interface hepatitis. Vascular dilatation was still seen. Foci of lobular mononuclear infiltrates were still detected in most of hepatic lobules.

Adding spiramycin and propolis to CS/Alg NP improved their anti-toxoplasma effect compared to crude drugs alone. Hepatic capsule did not show exudative changes. Moderate to minimal inflammation was still noted in some portal tracts but without interface hepatitis. Vascular dilatation was moderate. Foci of lobular infiltrate were small and infrequently seen.

The combined effect of spiramycin/propolis loaded CS/Alg NPs preparation showed the best effect in toxoplasmosis treatment. No capsular oedema was seen. Portal inflammation was focal and minimal. Vascular dilatations were minimal. Foci of lobular mononuclear infiltrate were only occasionally seen, and all were small sized with hardly spotted parasites.

#### II.4.B. Spleen (Fig 9)

As regards splenic histopathological examination, similar findings were seen. Infected untreated models showed perisplenitis with capsular inflammatory exudate and severely congested red pulp. The latter showed numerous histiocytes and megakaryocytes with numerous intra and extra cellular parasites. Mild improvement was noticed in the red pulp congestion as well as the megakaryocytic count for the groups receiving spiramycin, propolis and CS/Alg NPs. Adding CS/Alg NPs improved the effect of both spiramycin and propolis but congestion was still seen. Best results were seen in the group treated with spiramycin/propolis loaded CS/Alg NPs preparation. The congestion was minimal. Megakryoctes and histiocytes were rarely seen.

**Fig 9:**
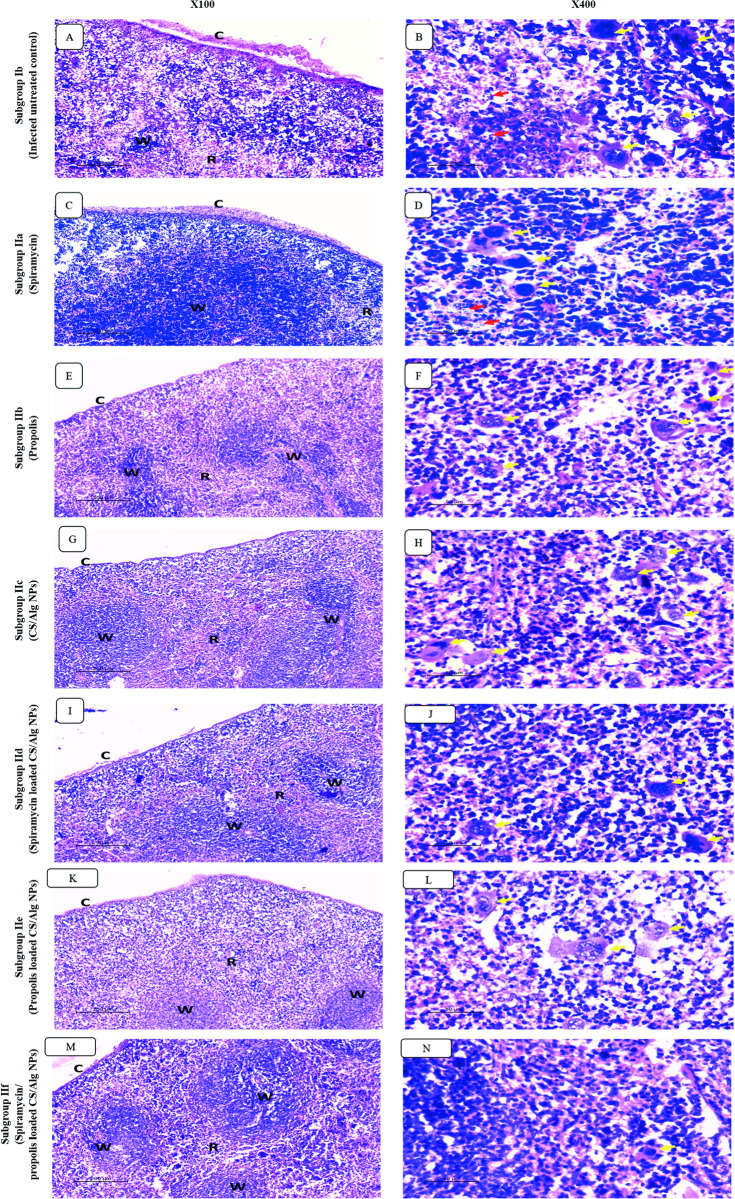
Sections of splenic tissues in infected untreated group (A-B) and treated groups with different regimens (C-N). (A) Infected untreated group showing perisplenitis (c) and severe congestion of red pulp (R) which occupied most of splenic architecture in expanse of white pulp (W). (B) High power view showing increased number of histiocytes and megakaryocytes (yellow arrows) with numerous parasites (red arrow) in infected untreated group. (C-N) Perisplenitis (C), red pulp congestion (R) and the count of megakaryocytes/histiocytes (yellow arrows) improved in different degrees in the treated groups.

#### II.4.C. Brain (Fig 10)

Brain sections of infected untreated group showed evident meningitis, parenchymal mononuclear infiltrates and diffuse degenerative neuronal changes as well as perivascular oedema. Those changes were still seen in spiramycin, propolis and CS/Alg NPs treated groups. Minimal improvement was seen after adding CS/Alg NPs to both drugs. Meanwhile the combined treatment by spiramycin/propolis loaded CS/Alg NPs preparation improved the histopathology of brain tissue. Meningitis was resolved with no mononuclear infiltration seen. Only focal neurodegenerative changes were seen.

**Fig 10:**
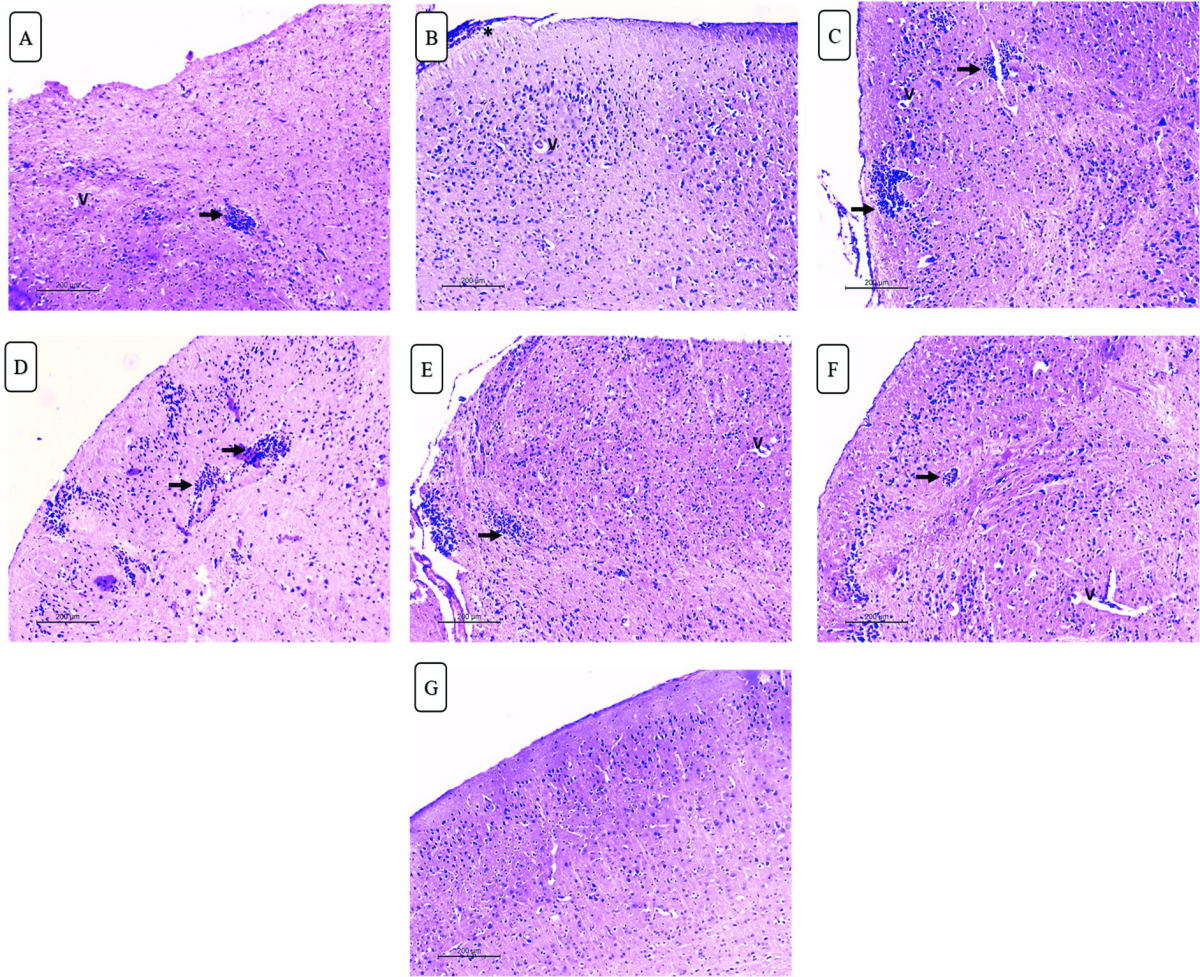
H&E-stained brain sections of different studied groups. (A) Infected untreated group showing mononuclear parenchymal infiltrate (black arrow) with perivascular oedema (v) (X100). (B) Mild improvement is seen in spiramycin treated group showing residual meningitis (asterisk) and perivascular oedema. (X100). (C-F) Mononuclear parenchymal infiltrate (black arrow) and perivascular oedema (v) are still noted in propolis, CS/Alg NPs, spiramycin CS/Alg NPs and propolis CS/Alg NPs treated groups respectively (X100). (G) Best results are seen in spiramycin/ propolis loaded CS/Alg NPs treated group with normal neurons, no meningitis or mononuclear infiltrate (X100).

## Discussion

*T*. *gondii* is one of the foremost successful parasites in the world with a wide range of hosts. Nearly half of the human population is infected by this parasite even without any symptoms. Tachyzoites of this parasite can cross the BBB causing fatal encephalitis within seconds, making its treatment a true challenge for many researchers. In this respect, the search for novel therapeutic regimens should be critically tracked to find an efficient and well-tolerated treatment [5,27]. Many studies were conducted using NPs as drug or vaccine vehicles to improve their therapeutic efficacy [26].

Several researches are interested in combining natural compounds with commonly known drugs to widen their biological activity [31]. However, propolis encapsulation in combined formula as anti-parasitic agent is scarce.

The main objective of the present study was to synthesize a novel nanocarrier (CS/Alg) to deliver the loaded drug (spiramycin) combined with propolis in a nanometric size to increase the surface area, bioavailability, biological activity and also enhance the tissue permeability of the drug.

In the present study, NPs characterization revealed smooth particles in the nano range with an increase of size upon drug loading from 20 nm to a maximum of 67.3 nm upon the loading of spiramycin/propolis on CS/Alg NPs. The resultant sizes of all the prepared formulae potentiate the tissue bypass. The smaller nanoparticles (< 200 nm) are less toxic in vivo with better tissue distribution, while larger nanoparticles (> 200 nm) are accumulated in the liver and spleen tissues [32]. CS/Alg NPs had negatively charged ζ potential. This could be explained by that the alginate dissolving occurs in a neutral pH which results in a negatively charged carboxylate groups [33]. However, all the prepared NPs had positively charged ζ potential which may be resulted from the formed hydrogen bond between amino and hydroxyl groups with the hydroxyl groups or oxygen atoms of water [34]. Another possible explanation of the charge changing from negative to positive was due to the mixing between the CS and Alg polysaccharides to form discrete NPs where part of the negatively charged Alg polysaccharide chains was buried in the core of the CS/Alg NPs [35]. The resulted positive charge was considered as an added value of the prepared NPs because this would enhance the NPs ability to transfer easily through the negative channels of the cell membrane [36]. The higher ζ potential was in accordance with the lower vesicle size and improved entrapment efficiency compared to CS/Alg NPs. It was stated that the higher ζ potential of the prepared nanoparticles, the less aggregation occurs. It was also mentioned that the NPs stability was improved by increasing the ζ potential higher than ± 30 mv [37].

As regards the clinical picture of the treated mice, this study showed improvement in the clinical presentation of mice infected with RH strain in the form of increase in food intake and activity in the cage in comparison to the infected untreated group. Noteworthy, the improvement was clear in those treated with the combination of spiramycin/propolis loaded CS/Alg NPs where mice appeared remarkably healthier than all other subgroups. This observation is in agreement with other studies [2,38].

In the context of the survival time and mortality rate on the sacrifice day, no survivors were observed beyond the 7^th^ day post-infection in infected untreated group. Similarly, other studies reported nearly the same results [24,27,38]. Remarkable statistically significant increase in the mean survival time was reported in all treated groups. Mice treated with spiramycin/propolis loaded CS/Alg NPs exhibited the longest mean survival time up to 19 days post-infection with no mortality at the day of sacrifice. In agreement with our results, other studies reported a significant increase in the survival time of mice treated with different nano-formulations [2,38]. Differences in mice survival time and mortality rates between the present study and others may be attributed to the variation in the mode of infection, parasite strain used, number of inoculated tachyzoites, route of infection, type of the used drug, different doses of drugs and their administration mode [2,38].

Estimation of the parasite load in different vital organs as liver, spleen and brain demonstrates the severity of infection and could roughly estimate the efficacy of the treatment [38]. In the present work, Giemsa-stained impression smears revealed highly significant pronounced reduction in parasite load in different organs especially those treated with spiramycin/propolis loaded CS/Alg NPs. A previous study used the closely related spiramycin loaded on CS NPs and showed a significant reduction in parasite count in different examined organs [2]. Recently, nitazoxanide loaded on silver nanoparticles exhibited a significant inhibitory effect on a chronic murine model of toxoplasmosis by reducing the number of brain tissue cysts [39].

Results of parasite burden in the present study were in agreement with the ultrastructure study of tachyzoites collected from infected mice. Most of the examined treated tachyzoites showed ulceration, furrows and were even ruptured especially in mice treated with spiramycin/propolis loaded CS/Alg NPs. Similarly, other studies reported that tachyzoites collected from treated mice showed marked morphological changes in the form of reduction in tachyzoite size with obvious surface irregularities and abnormal protrusion after treatment [26,40].

To confirm the achieved results, histopathological examination of liver, spleen and brain tissues of all studied groups was done. The histopathological study revealed that the toxoplasma tachyzoites load was markedly decreased in liver, spleen and brain sections among all treated groups in comparison to the infected untreated control. The least parasite load was obtained after the treatment with spiramycin/propolis loaded CS/Alg NPs compared to the other groups regarding all the studied organs. Severe degree of cellular damage was remarkable in the liver, spleen and brain tissues of acutely RH infected untreated mice. This could be explained by the ability of tachyzoites to invade any cell causing its rupture in addition to the powerful stimulation of inflammatory immune response with triggering of cytokines release causing lytic effect on the invaded cells [41,42].

As regards hepatic tissue examination of infected untreated control, the present results showed moderate to severe inflammatory infiltrates with interface hepatitis. The infiltrates were composed mainly of lymphocytes and plasma cells. Marked vascular dilatation and congestion were seen in all hepatic vessels including central and portal veins as well as sinusoids. Large foci of lobular mononuclear infiltrates were spotted frequently and with numerous intra and extra hepatocellular parasites. These findings agreed with those described by other authors [41]. Marked improvement of hepatic tissue was observed in mice treated with spiramycin/propolis loaded CS/Alg NPs where similar results were obtained by Allam et al. (2021) after treating mice with spiramycin-CS NPs in which the liver tissue showed evidence of regenerative hepatocytes with minimal dilatation of central veins and sinusoids in addition to minimal inflammation formed [5].

Regarding the spleen tissue of the infected untreated group, examination showed perisplenitis with capsular inflammatory exudate and severely congested red pulp. The latter showed numerous histiocytes and megakaryocytes with numerous intra and extracellular parasites. Megakaryocytosis and congested red pulp may be due to elevated level of IL12 which is released from stimulated inflammatory cells by tachyzoites of *T*. *gondii* as reported by Corrêa et al. (2017) [43].

Regarding the brain tissue examination, evident meningitis, parenchymal mononuclear infiltrates and diffuse degenerative neuronal changes as well as perivascular oedema were evident in mice infected and untreated. Similar observations were reported by other studies [5,42]. Spiramycin/propolis loaded CS/Alg NPs preparation improved the histopathology of brain tissue. Meningitis was resolved with no mononuclear infiltration seen. Only focal neurodegenerative changes were seen. Similar results were obtained by Allam et al. (2021) where spiramycin-CS NPs greatly reduced the inflammation and dead neurons were mineralized and replaced by gliotic nodules [5].

Histopathological study showed an overall marked improvement of the pathological pictures of liver, spleen and brain in the subgroup treated with spiramycin/propolis loaded CS/Alg NPs as opposed to other groups.

The observed results proved the efficacy of the prepared NPs in attribution to their small vesicle size which facilitates the drug distribution and absorption to target tissues, protect the drug from denaturation and degradation, increase the drug solubility and gradually release the drug [44].

## Conclusion

In conclusion, the current study revealed the powerful synergistic activity of the novel spiramycin/propolis loaded CS/Alg NPs which succeeded in tissue penetration and blood brain barrier passage. This in turn, provides an encouraging platform for the treatment of the virulent toxoplasma infection relying on the anti-parasitic and safe nature of each of spiramycin, propolis, CS and Alg.
